# Global climatological dataset of undersea acoustic parameters derived from the NCEI World Ocean Atlas 2023

**DOI:** 10.1038/s41597-024-04074-6

**Published:** 2024-11-28

**Authors:** Peter C. Chu, Chenwu Fan

**Affiliations:** Department of Oceanography, Naval Postgraduate School, Monterey, CA 93943 USA

**Keywords:** Ocean sciences, Physical oceanography

## Abstract

Acoustics is most effective in undersea detection, localization, and communication. Establishment of a global climatological dataset of undersea acoustic parameters becomes urgent. In building such a dataset, first we use the Thermodynamic Equation of Seawater-2010 (TEOS-10) to calculate the sound speed (SS) from the gridded temperature and salinity fields of the NOAA/NCEI World Ocean Atlas 2023. Second, we determine the depth of overall minimum from SS profile as the deep sound channel (DSC) axis depth, the depth of overall maximum between the surface and DSC axis as the sonic layer depth (SLD), the depth of the local minimum between SLD and DSC axis as the second sound channel (SSC) depth, and the depth with the SS equalling the maximum SS as the critical depth. Third, we obtain the SS at the surface, SLD, DSC axis, and SSC axis. Fourth, we determine the other acoustic parameters such as In-layer gradient, below layer gradient, DSC strength, SSC strength, depth excess, and surface duct cut-off frequency. The dataset is publicly available.

## Background & Summary

**Fig. 1:**
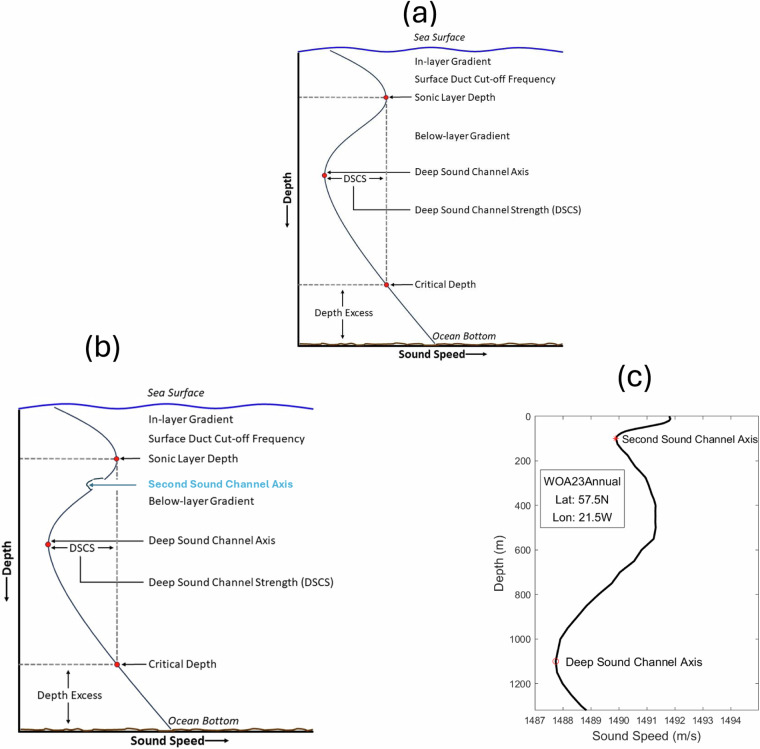
Illustration of a sound speed profile and definition of *D*_*dsc*_, *D*_*sl*_, *D*_*crit*_, *D*_*excess*_, in-layer gradient, below layer gradient, and DSC strength: (**a**) without second sound channel (typical), (**b**) with second sound channel (occasional), and (**c**) located at (21.5°W, 57.5° N) from the WOA23 annual data. These depths are defined in the Fleet Oceanographic and Acoustic Reference Manual by the Naval Oceanographic Office (2020). If a local minimum of the sound speed exists (not always exists) between *D*_*dsc*_ and *D*_*sl*_, the depth of the local minimum is defined as the second sound channel axis depth (*D*_*ssc*_).

Undersea acoustic parameters such as deep sound channel (DSC) axis depth, sonic layer depth (SLD), second (local) sound channel axis depth, critical depth, depth excess are fully determined from a typical sound speed profile (Fig. 1a). They are crucial to affect acoustic transmission and in turn impact acoustic detection, localization, and communication^1^. Sound speed depends on temperature, salinity, and depth. With given temperature and salinity profiles, the sound speed changes with depth. Let *c*(*z*) be the sound speed profile with z ≥ 0 (downward increasing), representing the vertical with *z* = 0 for the ocean surface and *z* = *H* for the bottom topography. The depth of overall minimum of *c*(*z*),

1 $${c}_{\min }=\mathop{\min }\limits_{H\ge z\ge 0}[c(z)],\,c({D}_{dsc})={c}_{\min }$$

is defined as the DSC axis depth (*D*_*dsc*_). The depth of overall maximum of *c*(*z*) between z = 0 and *z* = *D*_*dsc*_,

2 $${c}_{\max }=\mathop{\min }\limits_{{D}_{dsc}\ge z\ge 0}[c(z)],\,c({D}_{sl})={c}_{\max }$$

is defined as the sonic layer depth (*D*_*sl*_). The layer between *z* = 0 and *z* = *D*_*sl*_ is called the sonic layer (SL). If local minimum exists in c(z) between *D*_*sl*_ and *D*_*dsc*_ (Fig. 1b,c),

3 $${\hat{c}}_{\min }=\mathop{\min }\limits_{{D}_{dsc} > z > {D}_{sl}}[c(z)],\,c({D}_{ssc})={\hat{c}}_{\min }$$

which is obtained by,

4 $$\frac{\partial c}{\partial z}{|}_{z={D}_{ssc}}=0,\,\frac{{\partial }^{2}c}{\partial {z}^{2}}{|}_{z={D}_{ssc}} > 0,\,{\rm{for}}\,{D}_{dsc} > z > {D}_{sl}$$

The depth of the local minimum (*D*_*ssc*_) is the second (or local) sound channel axis depth. The second sound channel occurs occasionally; however, it enhances the acoustic transmission. Below z = *D*_*dsc*_, the depth with the sound speed equals *c*_max_ is called the critical depth (*D*_*crit*_),

5 $$c({D}_{crit})={c}_{\max },\,{\rm{for}}\,z > {D}_{dsc}$$

The difference between the bottom topography and the critical depth,

6 $${D}_{excess}=H-{D}_{crit}$$

is called the depth excess (*D*_*excess*_).

With these depths and corresponding sound speeds at the depths, other acoustic parameters such as the deep sound channel strength, in layer gradient, below layer gradient, and surface duct cutoff frequency can be determined.

There is no publicly available climatological dataset for these acoustic parameters despite they are important for undersea acoustic detection, localization, and communication and no matter the climatological sound speed data can be calculated from the climatological (*T*, *S*) datasets. To fill the gap, we compute the climatological annual and seasonal mean world ocean sound speed using the NOAA/National Centers for Environmental Information (NCEI) ‘s World Ocean Atlas (WOA) 2023 (T, S) data^2–4^ and then obtain the world ocean climatological annual and seasonal mean 13 undersea acoustic parameters dataset with 5°, 1°, and 0.25° horizontal resolutions which has been published at the NOAA/NCEI website for public use (https://www.ncei.noaa.gov/archive/accession/0290599).

## Methods

### Use of existing dataset

We produce this dataset based on the existing data. The NOAA/NCEI WOA 2023 (WOA23) annual and seasonal mean temperature and salinity profiles^2–4^ at regular 102 vertical levels (Table 1) are used to calculate the sound speed *c*(*z*) at the WOA23 horizontal grid points with the Thermodynamic Equation of Saewater-2010 (TEOS-10) (https://www.teos-10.org/). Here, the MATLAB code gsw_sound_speed.m is used.

**Table 1:** Standard vertical depths of WOA23 data.

Standard Level	Standard Depth (m)	Standard Level	Standard Depth (m)	Standard Level	Standard Depth (m)	Standard Level	Standard Depth (m)
1	0	27	250	53	1300	79	3200
2	5	28	275	54	1350	80	3300
3	10	29	300	55	1400	81	3400
4	15	30	325	56	1450	82	3500
5	20	31	350	57	1500	83	3600
6	25	32	375	58	1550	84	3700
7	30	33	400	59	1600	85	3800
8	35	34	425	60	1650	86	3900
9	40	35	450	61	1700	87	4000
10	45	36	475	62	1750	88	4100
11	50	37	500	63	1800	89	4200
12	55	38	550	64	1850	90	4300
13	60	39	600	65	1900	91	4400
14	65	40	650	66	1950	92	4500
15	70	41	700	67	2000	93	4600
16	75	42	750	68	2100	94	4700
17	80	43	800	69	2200	95	4800
18	85	44	850	70	2300	96	4900
19	90	45	900	71	2400	97	5000
20	95	46	950	72	2500	98	5100
21	100	47	1000	73	2600	99	5200
22	125	48	1050	74	2700	100	5300
23	150	49	1100	75	2800	101	5400
24	175	50	1150	76	2900	102	5500
25	200	51	1200	77	3000		
26	225	52	1250	78	3100		

Global climatological (annual mean and seasonal mean) data of undersea acoustic parameters can be established through two approaches: (1) analyzing climatological sound speed profiles, and (2) analyzing observational sound speed profiles to get synoptic under water acoustic parameters and then using the optimal interpolation^5^, Kalmen filter^6^, or optimal spectral decomposition^7^ to produce gridded climatological underwater acoustic parameters. At present, we cannot estimate how big the difference is between using these two approaches. We can estimate only after taking the two approaches. In this study, we take the first approach to derive climatological underwater acoustic parameter data from the climatological sound speed profiles calculated from the NOAA/NCEI World Ocean Atlas 2023 (WOA23)^2–4^ annual and seasonal mean temperature and salinity (*S*) profiles with regular 102 vertical levels (Table 1).

### Determination of five depths (D_dsc_, D_sl_, D_ssc_, D_crit_, D_excess_)

Let a sound speed profile at the WOA23 horizontal grid point starting from *z*_1_ at the surface to any depth *z*_*k*_ (shown in Table 1) be represented by *c*(*z*_*k*_). Here, *k* = 1, 2, …, K, with *z*_*K*_ the bottom of the profile. The depth of overall minimum of *c*(*z*_*k*_) is obtained from Eq. (1) to identify the DSC axis depth (*D*_*dsc*_). The DSC is often referred to as Sound Fixing and Ranging (SOFAR) channel, where the sound waves are effectively confined and trace a path that oscillates across the DSC axis. The DSC allows sound to carry great distance^8^.

**Fig. 2:**
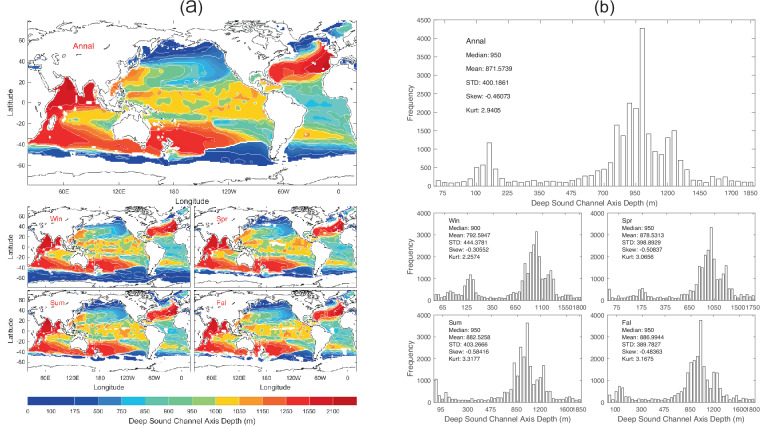
(**a**) Maps and (**b**) histograms of global climatological annual and seasonal mean data with 1° resolution of *D*_*dsc*_ (unit: m).

The climatological sound speed profiles calculated from the WOA23 temperature and salinity profiles are not exactly follow the conceptual profile described in Fig. 1a. Therefore, the dataset of 13 undersea acoustic parameters does not appear to be global. It lacks coverage for the Arctic Ocean, the southern part of the Southern Ocean, and coastal seas. This is because that these sound speed profiles do not follow the profile depicted in Fig. 1a. After the 13 undersea acoustic parameters were identified, the statistical characteristics such as mean, standard deviation, skewness, and kurtosis were calculated using the MATLAB functions for each acoustic parameter over the global oceans. Data of *D*_*dsc*_ with 1° horizontal resolution are presented with annual and seasonal mean maps (Fig. 2a) and histograms (Fig. 2b). *D*_*dsc*_ has evident spatial variability but minor seasonal variation (Fig. 2a) with median (950 m), mean (871.6 m), and standard deviation (400.2 m) of the annual mean data (Fig. 2b).

**Fig. 3:**
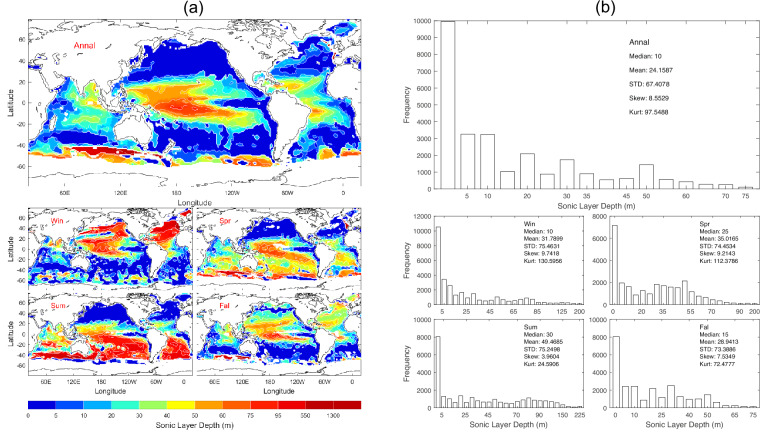
(**a**) Maps and (**b**) histograms of global climatological annual and seasonal mean data with 1° resolution of *D*_*sl*_ (unit: m).

The depth of overall maximum of *c*(*z*_*k*_) between z_1_ and *D*_*dsc*_ is identified as the SLD (*D*_*sl*_) [see Eq. (2)], which represents the bottom limit of the surface duct^9^, a layer where sound waves are refracted back towards the surface, facilitating longer horizontal travel. The SLD also marks the upper boundary of the DSC. Understanding the SLD is crucial for effective sonar operation, undersea communication, and acoustic propagation. Data of *D*_*sl*_ with 1° horizontal resolution are presented with annual and seasonal mean maps (Fig. 3a) and histograms (Fig. 3b). *D*_*sl*_ has evident spatial variability and seasonal variation (Fig. 3a) with median (10 m), mean (24.2 m), and standard deviation (67.4 m) of the annual mean data (Fig. 3b).

**Fig. 4:**
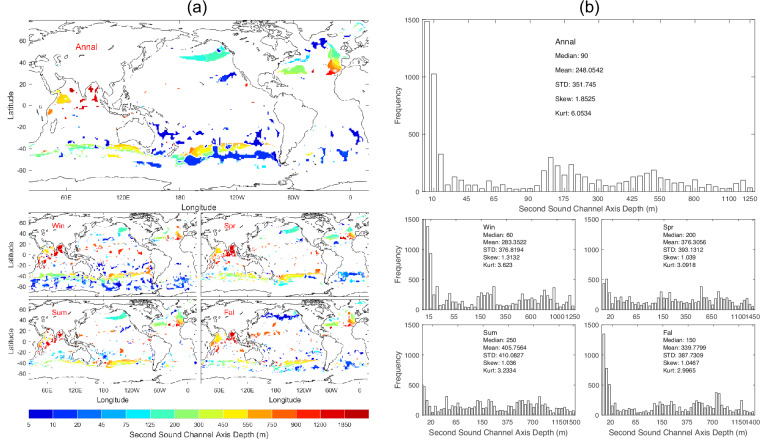
(**a**) Maps and (**b**) histograms of global climatological annual and seasonal mean data with 1° resolution of *D*_*ssc*_ (unit: m).

The second sound channel (SSC) axis depth (*D*_*ssc*_) is identified by the existence of local minimum in c(z_k_) between *D*_sl_ and *D*_*dsc*_ [see Eq. (4)]. Data of *D*_*ssc*_ with 1° horizontal resolution are presented with annual and seasonal mean maps (Fig. 4a) and histograms (Fig. 4b). The SSC exists sporadically (rare occurrence) and varies seasonally (Fig. 4a) with median (125 m), mean (216.8 m), and standard deviation (231.2 m) of the annual mean data (Fig. 4b).

**Fig. 5:**
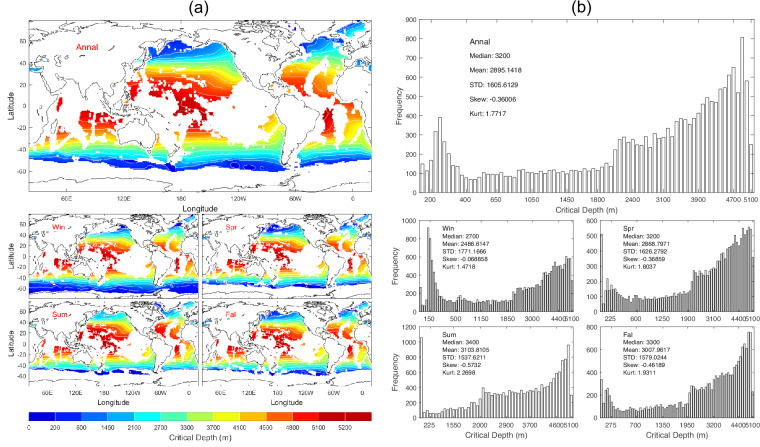
(**a**) Maps and (b) histograms of global climatological annual and seasonal mean data with 1° resolution of *D*_*crit*_ (unit: m).

The critical depth (*D*_*crit*_) is identified as the specific depth beneath the DSC axis where the sound speed equals that at *D*_*s*_^1^ [see Eqs. 1, 5]. This depth is pivotal as it signifies the lower boundary of the DSC. As one moves deeper past the DSC axis, the sound speed begins to increase and eventually to match the sound speed at the SLD. It marks a significant transition in the undersea acoustic environment, indicating a change in sound propagation beyond *D*_*crit*_. Data of *D*_*crit*_ with 1° horizontal resolution are presented with annual and seasonal mean maps (Fig. 5a) and histograms (Fig. 5b). *D*_*crit*_ has evident spatial variability but minor seasonal variation (Fig. 5a) with median (3200 m), mean (2895.1 m), and standard deviation (1605.6 m) of the annual mean data (Fig. 5b).

**Fig. 6:**
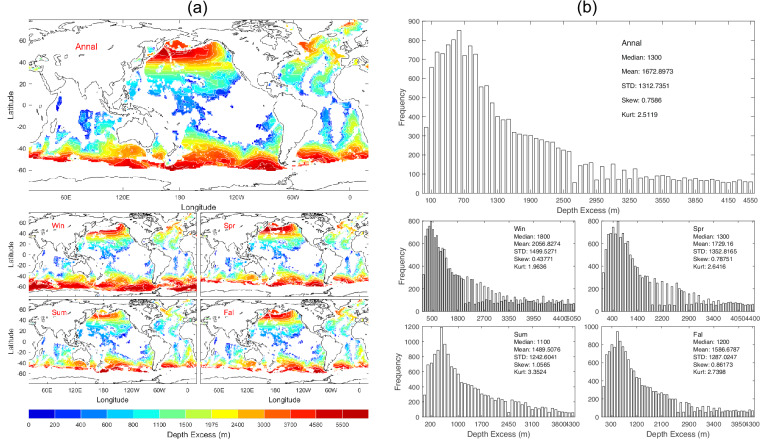
(**a**) Maps and (**b**) histograms of global climatological annual and seasonal mean data with 1° resolution of *D*_*excess*_ (unit: m).

Correspondingly, the depth excess (*D*_*excess*_) is obtained using Eq. (6). Data of *D*_*excess*_ with 1° horizontal resolution are presented with annual and seasonal mean maps (Fig. 6a) and histograms (Fig. 6b). *D*_*excess*_ has evident spatial variability but minor seasonal variation (Fig. 6a) with median (1300 m), mean (1672.9 m), and standard deviation (1312.7 m) of the annual mean data (Fig. 6b).

### Sound speeds at surface and (D_s_, D_dsc_, D_ssc_,)

The sound speed at the ocean surface is represented by,

7 $${c}_{0}={c}_{|z=0}.$$

**Fig. 7:**
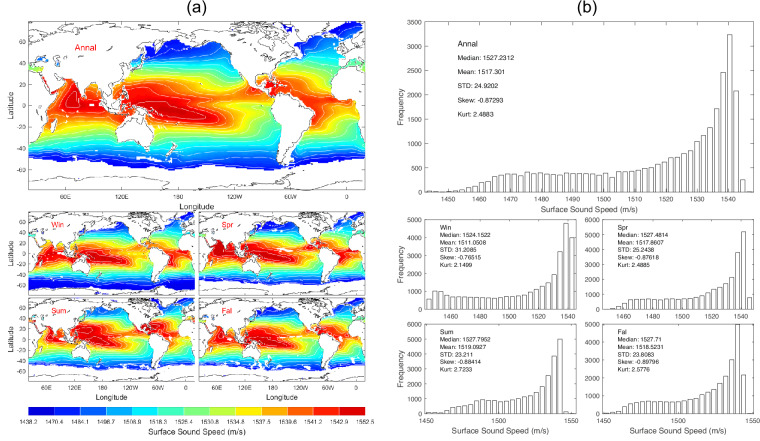
(**a**) Maps and (**b**) histograms of global climatological annual and seasonal mean data with 1° resolution of *c*_0_ (unit: m s^−1^).

Data of *c*_0_ with 1° horizontal resolution are presented with annual and seasonal mean maps (Fig. 7a) and histograms (Fig. 7b). *c*_0_ has evident spatial variability but minor seasonal variation (Fig. 7a) with median (1527.2 m/s), mean (1517.3 m/s), and standard deviation (24.92 m/s) of the annual mean data (Fig. 7b).

The sound speed at the SLD is given by,

8 $${c}_{sld}=c({D}_{sl})={c}_{max}.$$

**Fig. 8:**
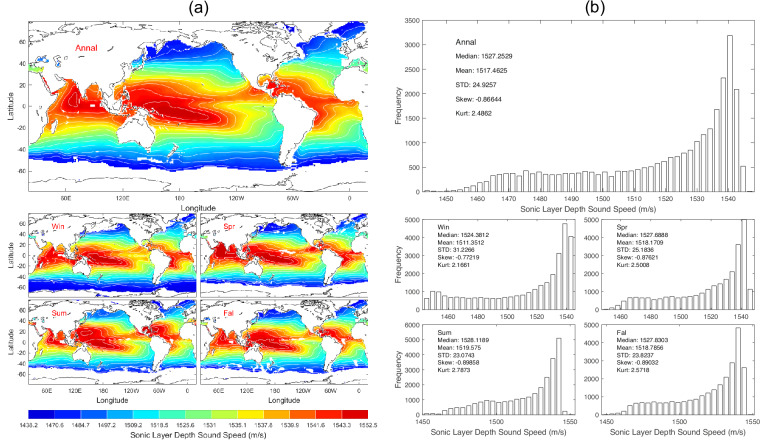
(**a**) Maps and (**b**) histograms of global climatological annual and seasonal mean data with 1° resolution of *c*_*sld*_ (unit: m s^−1^).

Data of *c*_*sld*_ with 1° horizontal resolution are presented with annual and seasonal mean maps (Fig. 8a) and histograms (Fig. 8b). *c*_*sld*_ has evident spatial variability but minor seasonal variation (Fig. 8a) with median (1527.3 m/s), mean (1517.5 m/s), and standard deviation (24.93 m/s) of the annual mean data (Fig. 8b).

The sound speed at the DSC axis is defined by,

9 $${c}_{dsc}=c({D}_{dsc})={c}_{\min }.$$

**Fig. 9:**
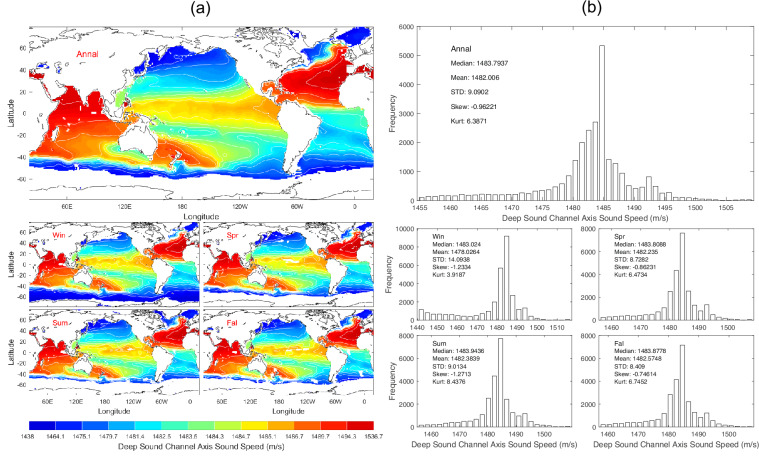
(**a**) Maps and (**b**) histograms of global climatological annual and seasonal mean data with 1° resolution of *c*_*dsc*_ (unit: m s^−1^).

Data of *c*_*dsc*_ with 1° horizontal resolution are presented with annual and seasonal mean maps (Fig. 9a) and histograms (Fig. 9b). *c*_*dsc*_ has evident spatial variability but minor seasonal variation (Fig. 9a) with median (1483.8 m/s), mean (1482.0 m/s), and standard deviation (9.09 m/s) of the annual mean data (Fig. 9b).

### DSC and SSC strengths

The deep sound channel strength (*S*_*dsc*_) is defined by,

10 $${S}_{dsc}={c}_{\max }-{c}_{\min }.$$

**Fig. 10:**
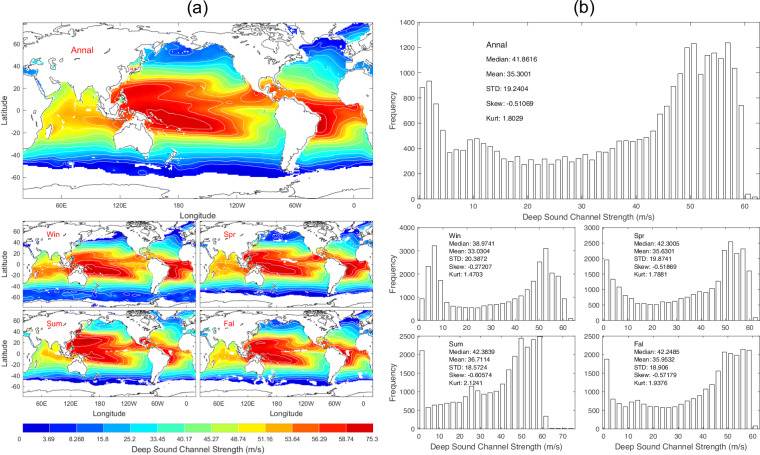
(**a**) Maps and (**b**) histograms of global climatological annual and seasonal mean data with 1° resolution of *S*_*dsc*_ (unit: m s^−1^).

Data of S_*dsc*_ with 1° horizontal resolution are presented with annual and seasonal mean maps (Fig. 10a) and histograms (Fig. 10b). *S*_*dsc*_ has evident spatial variability but minor seasonal variation (Fig. 10a) with median (41.86 m/s), mean (35.30 m/s), and standard deviation (19.24 m/s) of the annual mean data (Fig. 10b).

The SSC strength (*S*_*ssc*_) is defined by,

11 $${S}_{ssc}={c}_{\max }-{\hat{c}}_{\min }.$$

**Fig. 11:**
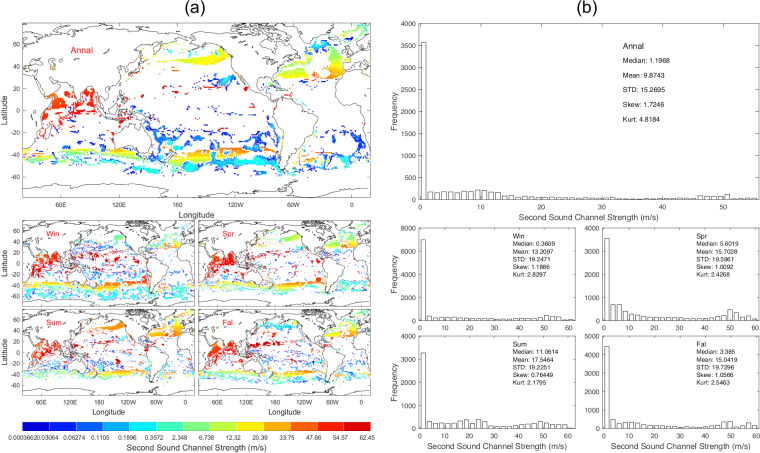
(**a**) Maps and (**b**) histograms of global climatological annual and seasonal mean data with 1° resolution of *S*_*ssc*_ (unit: m s^−1^).

Data of *S*_*ssc*_ with 1° horizontal resolution are presented with annual and seasonal mean maps (Fig. 11a) and histograms (Fig. 11b). The *S*_*ssc*_ exists sporadically and varies seasonally (Fig. 11a) with median (6.51 m/s), mean (9.70 m/s), and standard deviation (11.75 m/s) of the annual mean data (Fig. 11b).

### In-layer and below layer gradients

The in-layer gradient is defined by,

12 $${G}_{in}=({c}_{d}-{c}_{0})/{D}_{sl}$$

**Fig. 12:**
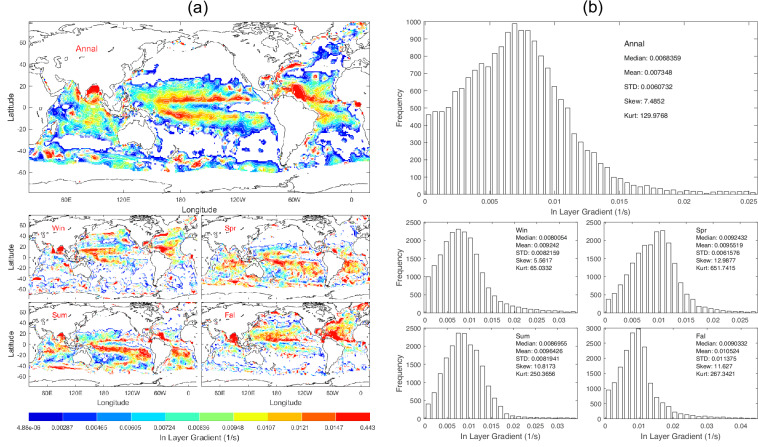
(**a**) Maps and (**b**) histograms of global climatological annual and seasonal mean data with 1° resolution of *G*_*in*_ (unit: s^−1^).

Data of *G*_*in*_ with 1° horizontal resolution are presented with annual and seasonal mean maps (Fig. 12a) and histograms (Fig. 12b). *G*_*in*_ has evident spatial variability and seasonal variation (Fig. 12a) with median (6.836 × 10^−3^ s^−1^), mean (7.348 × 10^−3^ s^−1^), and standard deviation (6.073 × 10^−3^ s^−1^) of the annual mean data (Fig. 12b).

**Fig. 13:**
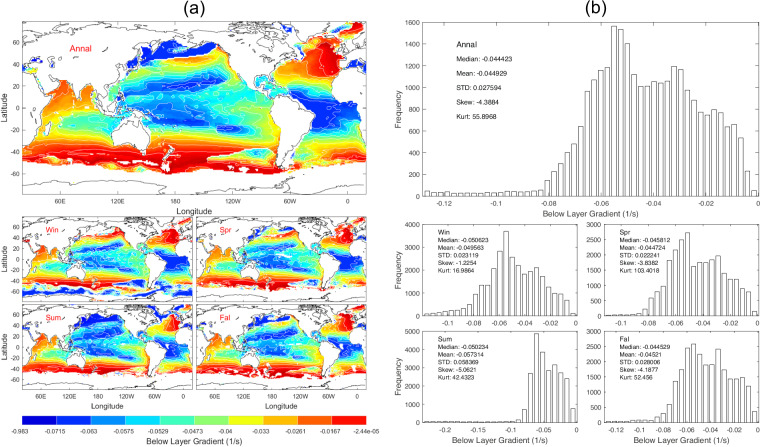
(**a**) Maps and (**b**) histograms of global climatological annual and seasonal mean data with 1° resolution of *G*_*below*_ (unit: s^−1^).

The below layer gradient (*G*_*below*_) is the mean vertical gradient between *D*_*sl*_ and *D*_*dsc*_. Data of *G*_*below*_ with 1° horizontal resolution are presented with annual and seasonal mean maps (Fig. 13a) and histograms (Fig. 13b). *G*_*below*_ has evident spatial variability and seasonal variation (Fig. 13a) with median (−44.42 × 10^−3^ s^−1^), mean (−44.93 × 10^−3^ s^−1^), and standard deviation (27.59 × 10^−3^ s^−1^) of the annual mean data (Fig. 13b).

### Surface duct cutoff frequency

At very low frequencies, sound ceases to be trapped in the mixed layer or, in any sound channels. This occurs when the frequency for the first mode of normal mode theory, when, in a sense, the wavelength has become too large to ‘fit’ in the duct. This maximum wavelength (λ_max_) for duct transmission may be found from the theory of radio propagation in ground-based radio ducts. Using values of sound speed and in-layer gradient for sound transmission in the isothermal layer, the maximum trapped wavelength λ_max_ is represented by^9^,

13 $${\lambda }_{\max }=8.513\times {10}^{-3}{D}^{3/2},\,({\rm{unit}}:\,{\rm{m}})\,{\rm{for}}\,({\lambda }_{\max },\,D)$$

where *D* is the isothermal layer depth (in meter). For *D* = 30.48 m (100 ft), the maximum trapped wavelength λ_max_ is 1.43 m (4.7 ft). The surface duct cutoff frequency (*f*_*cutoff*_) is represented by,

14 $${f}_{cutoff}=\frac{{c}_{0}}{{\lambda }_{\max }}$$

Substitution of Eqs. (13, 14) leads to

15 $${f}_{cutoff}=\frac{{c}_{0}}{8.513}\times {10}^{3}{D}^{-3/2}\,({\rm{unit}}:\,{\rm{Hz}})$$

The isothermal layer depth (*D*) was identified from WOA23 temperature data using the exponential leap-forward gradient method described in the reference^10^ and published at the NOAA/NCEI website^11,12^.

**Fig. 14:**
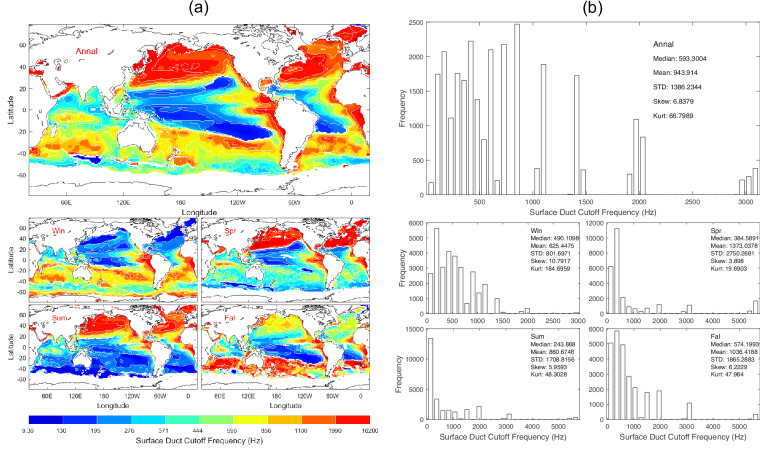
(**a**) Maps and (**b**) histograms of global climatological annual and seasonal mean data with 1° resolution of *f*_*cutoff*_ (unit: Hz).

Figure 14 shows the annual and seasonal means of the surface duct cutoff frequency. Data of *f*_*cutoff*_ with 1° horizontal resolution are presented with annual and seasonal mean maps (Fig. 14a) and histograms (Fig. 14b). *f*_*cutoff*_ has evident spatial variability and seasonal variation (Fig. 14a) with median (593 Hz), mean (944 Hz), and standard deviation (1386 Hz) of the annual mean data (Fig. 14b). Since in Eq.(15) the isothermal layer depth (*D*) identified from WOA23 temperature data takes discrete WOA23 standard depths^12^ as shown in Table 1, and the surface sound speed varies from 1,517 m s^−1^ mildly, *f*_cutoff_ may take discrete values. This causes gaps in the histograms of *f*_cutoff_ as shown in Fig. 14b.

### Statistical characteristics

We calculated the median, mean, standard deviation, skewness, and kurtosis of the thirteen parameters for the annual mean (Table 2), winter (Table 3), spring (Table 4), summer (Table 5), and fall (Table 6). These values can also be found in Figs. 2b, 14b.

**Table 2:** Median, mean, and standard deviation the global annual mean undersea acoustic parameters.

Parameter	Median	Mean	Standard Deviation	Skewness	Kurtosis
Deep Sound Channel Axis Depth (m)	950.0	871.6	400.2	−0.46	2.94
Sonic Layer Depth (m)	10.0	24.2	67.4	8.55	97.55
Second Sound Channel Axis Depth (m)	125.0	216.8	231.2	1.36	4.83
Critical Depth (m)	3200.0	2895.1	1605.6	−0.36	1.77
Depth Excess (m)	1300.0	1672.9	1312.7	0.76	2.51
Surface Sound Speed (m/s)	1527.2	1517.3	24.9	−0.87	2.49
Sonic Layer Depth Sound Speed (m/s)	1527.3	1517.5	24.9	−0.87	2.49
Deep Sound Channel Axis Sound Speed (m/s)	1483.8	1482.0	9.1	−0.96	6.39
Deep Sound Channel Strength (m/s)	41.86	35.30	19.24	−0.51	1.80
Second Sound Channel Strength (m/s)	6.51	9.70	11.75	1.66	5.48
In-Layer Gradient (10^−3^ 1/s)	6.836	7.348	6.073	7.49	129.98
Below Layer Gradient (10^−3^ 1/s)	−44.42	−44.93	27.59	−4.39	55.90
Surface Duct Cutoff Frequency (Hz)	593	944	1386	6.84	66.80

**Table 3:** Median, mean, and standard deviation the global winter mean undersea acoustic parameters.

Parameter	Median	Mean	Standard Deviation	Skewness	Kurtosis
Deep Sound Channel Axis Depth (m)	900.0	792.6	444.4	−0.31	2.26
Sonic Layer Depth (m)	10.0	31.8	75.5	9.74	130.6
Second Sound Channel Axis Depth (m)	45.0	172.9	233.6	1.95	7.60
Critical Depth (m)	2700.0	2486.6	1771.2	−0.07	1.47
Depth Excess (m)	1800.0	2056.8	1499.5	0.44	1.96
Surface Sound Speed (m/s)	1524.2	1511.1	31.21	−0.77	2.15
Sonic Layer Depth Sound Speed (m/s)	1524.4	1511.4	31.20	−0.77	2.17
Deep Sound Channel Axis Sound Speed (m/s)	1483.0	1478.0	14.10	−1.23	3.92
Deep Sound Channel Strength (m/s)	38.97	33.03	20.39	−0.27	1.47
Second Sound Channel Strength (m/s)	0.56	9.43	14.00	1.56	4.36
In-Layer Gradient (10^−3^ 1/s)	8.005	9.242	8.216	5.56	65.03
Below Layer Gradient (10^−3^ 1/s)	−50.62	−49.56	23.12	−1.23	16.99
Surface Duct Cutoff Frequency (Hz)	490	625	802	10.79	184.70

**Table 4:** Median, mean, and standard deviation the global spring mean undersea acoustic parameters.

Parameter	Median	Mean	Standard Deviation	Skewness	Kurtosis
Deep Sound Channel Axis Depth (m)	950.0	878.5	398.9	−0.51	3.07
Sonic Layer Depth (m)	25.0	35.0	74.5	9.21	112.4
Second Sound Channel Axis Depth (m)	95.0	182.3	249.7	2.44	9.20
Critical Depth (m)	3200.0	2868.8	1626.3	−0.37	1.80
Depth Excess (m)	1300.0	1729.2	1352.8	0.79	2.64
Surface Sound Speed (m/s)	1527.5	1517.9	25.2	−0.88	2.49
Sonic Layer Depth Sound Speed (m/s)	1527.7	1518.2	25.2	−0.88	2.50
Deep Sound Channel Axis Sound Speed (m/s)	1483.8	1482.2	8.73	−0.86	6.47
Deep Sound Channel Strength (m/s)	42.30	35.63	19.87	−0.52	1.79
Second Sound Channel Strength (m/s)	3.50	8.92	13.39	1.94	5.88
In-Layer Gradient (10^−3^ 1/s)	9.243	9.552	6.158	12.99	651.74
Below Layer Gradient (10^−3^ 1/s)	−45.81	−44.72	22.24	−3.84	103.40
Surface Duct Cutoff Frequency (Hz)	385	1373	2750	3.90	19.69

**Table 5:** Median, mean, and standard deviation the global summer mean undersea acoustic parameters.

Parameter	Median	Mean	Standard Deviation	Skewness	Kurtosis
Deep Sound Channel Axis Depth (m)	950.0	882.5	403.3	−0.58	3.32
Sonic Layer Depth (m)	30.0	49.5	75.2	3.96	24.6
Second Sound Channel Axis Depth (m)	90.0	180.2	265.0	2.45	8.95
Critical Depth (m)	3400.0	3103.8	1537.6	−0.57	2.27
Depth Excess (m)	1100.0	1489.5	1242.6	1.06	3.35
Surface Sound Speed (m/s)	1527.8	1519.1	23.2	−0.88	2.72
Sonic Layer Depth Sound Speed (m/s)	1528.1	1519.6	23.1	−0.90	2.79
Deep Sound Channel Axis Sound Speed (m/s)	1483.9	1482.4	9.0	−1.27	8.44
Deep Sound Channel Strength (m/s)	42.38	36.71	18.57	−0.61	2.12
Second Sound Channel Strength (m/s)	1.32	11.40	14.53	1.10	3.17
In-Layer Gradient (10^−3^ 1/s)	8.696	9.643	8.194	10.82	250.37
Below Layer Gradient (10^−3^ 1/s)	−50.23	−57.31	58.37	−5.06	42.43
Surface Duct Cutoff Frequency (Hz)	244	861	1709	5.96	48.30

**Table 6:** Median, mean, and standard deviation the global fall mean undersea acoustic parameters.

Parameter	Median	Mean	Standard Deviation	Skewness	Kurtosis
Deep Sound Channel Axis Depth (m)	950.0	887.0	389.8	−0.48	3.17
Sonic Layer Depth (m)	15.0	28.9	73.4	7.53	72.48
Second Sound Channel Axis Depth (m)	100.0	230.8	265.5	1.62	6.26
Critical Depth (m)	3300.0	3008.0	1579.0	−0.46	1.93
Depth Excess (m)	1200.0	1586.7	1287.0	0.86	2.74
Surface Sound Speed (m/s)	1527.7	1518.5	23.8	−0.90	2.58
Sonic Layer Depth Sound Speed (m/s)	1527.8	1518.8	23.8	−0.89	2.57
Deep Sound Channel Axis Sound Speed (m/s)	1483.9	1482.6	8.4	−0.75	6.75
Deep Sound Channel Strength (m/s)	42.25	35.95	18.91	−0.57	1.94
Second Sound Channel Strength (m/s)	4.33	10.31	13.62	1.59	4.66
In-Layer Gradient (10^−3^ 1/s)	9.033	10.524	11.375	11.63	267.34
Below Layer Gradient (10^−3^ 1/s)	−44.53	−45.21	28.01	−4.19	52.46
Surface Duct Cutoff Frequency (Hz)	574	1036	1865	6.22	47.98

## Data Records

This global dataset for climatological annual and seasonal mean undersea acoustic parameters such as the sonic layer depth (SLD), deep sound channel (DSC) axis depth, second sound channel (SSC) axis depth, sound speed at the surface, sound speed at the SLD, sound speed at the DSC axis, sound speed at the SSC axis, in-layer gradient, below-layer gradient, surface duct cut-off frequency, deep sound channel strength, critical depth of the DSC, depth excess, and second sound channel strength has been established using the NOAA/NCEI WOA23 temperature and salinity data^2–4^ and derived isothermal layer depth (D) data^12^ (for surface duct cutoff frequency). This dataset^13^ is publicly available at the NOAA/NCEI data repository 10.25921/fe7c-5f76 as a NetCDF file, which includes data citation, dataset identifiers, metadata, and ordering instructions.

## Technical Validation

The uncertainty in determining the undersea acoustic parameters from a sound speed profile comes not only from the temperature data but also from the depth (pressure). This is because an observational temperature profile data has errors in the depth such as the XBT, MBT, and XCTD temperature profiles. The uncertainty in depth is caused by the not fully accurate drop-rate equation, which converts time since drop to depth in the ocean. After the drop-rate correction, WOA23 produces temperature profile data at 102 standard levels shown in Table 1 with uncertainty only from the temperature and salinity data and not from the depth^2,3^.

The thirteen undersea acoustic parameters are calculated from the WOA23 temperature and salinity profile data (i.e., sound speed profile data) using the analytical formulae (1)–(15). The uncertainty of the undersea acoustic parameter data is caused by the uncertainty in temperature and salinity data. The uncertainty of the WOA23 temperature is ±0.003 °C^2^. The effect of temperature on the undersea sound speed is much more than salinity. A random noise with intensity of 0.003 °C is added to each grid point of WOA23 temperature field. The perturbed undersea sound speed field and in turn the 13 perturbed undersea acoustic parameters are calculated. Let the unperturbed and perturbed acoustic parameters be represented by ψ_0_ and ψ. The root mean square of (ψ- ψ_0_) divided by the standard deviation of ψ_0_, (i.e., relative root-mean square error),

16 $$\mu =\frac{\sqrt{\frac{1}{M}\sum {(\psi -{\psi }_{0})}^{2}}}{STD({\psi }_{0})},$$

is used to represent the uncertainty of the derived undersea acoustic parameter dataset. The random noise (±0.003 °C) has negligible effect on sound speed at the surface (*μ* = 2.07 × 10^−4^), sonic layer depth (*μ* = 3.81 × 10^−4^), deep sound channel axis depth (*μ = *7.85 × 10^−4^), deep sound channel strength (*μ* = 4.57 × 10^−4^), and small effect on sonic layer depth (*μ* = 0.042), deep sound channel axis depth (*μ* = 0.037), second sound channel strength (*μ* = 0.076).

In addition, the uncertainty in the isothermal depth (*D*) may also introduce uncertainty in the surface duct cutoff frequency (*f*_*cutoff*_). The global climatological isothermal layer depth (*D*) data were produced using the exponential leap-forward gradient method^10^, which was developed on the base of several earlier schemes^14–16^. The relative error of the isothermal depth (D) using the exponential leap-forward gradient method is given by (see Table 3 in ref. ^10^)

17 $$\frac{|\delta D|}{D}\approx 0.02$$

Use of (15) and (17) leads to the relative error of the global surface duct frequency,

18 $$\frac{|\delta {f}_{cutoff}|}{{f}_{cutoff}}=\frac{3}{2}\frac{|\delta D|}{D}\approx 0.03.$$

## Data Availability

We use the MATLAB code gsw_sound_speed.m located at the website https://www.teos-10.org/ to compute sound speed profiles from the WOA23 annual and seasonal mean temperature and salinity profiles; and use the basic MATLAB functions related to Eqs. (1)–(15) to produce global climatological dataset of 13 acoustic parameters^13^. It is not necessary to present the code.

## References

[1] Naval Oceanographic Office. *Fleet Oceanographic and Acoustic Reference Manual*. Reference Publication PR33 (2020).

[2] Locarnini, R. A. *et al*. *World Ocean Atlas 2023, Volume 1: Temperature*. A. Mishonov Tech. Ed., NOAA Atlas NESDIS 89, 52 pp, 10.25923/54bh-1613 (2024).

[3] Reagan, J. R. *et al*. *World Ocean Atlas 2023 Volume 2: Salinity*. A. Mishonov, Tech. Ed., NOAA Atlas NESDIS 90, 51pp. 10.25923/70qt-9574 (2024).

[4] NOAA National Centers for Environmental Information. *World Ocean Atlas 2023*10.25923/54bh-1613 and 10.25923/70qt-9574 (2024).

[5] Bretherton, F. P., Davis, R. E. & Fandry, C. B. A technique for objective analysis and design of oceanographic experiments applied to MODE-73. *Deep-Sea Research* **23**, 559–582, 10.1016/0011-7471(76)90001-2 (1976).

[6] Evensen, G. The ensemble Kalman filter: theoretical formulation and practical implementation. *Ocean Dynamics* **53**, 343–367 (2003).

[7] Chu, P. C., Fan, C. W. & Margolina, T. Ocean spectral data assimilation without background error covariance matrix. *Ocean Dynamics* **66**, 1143–1163, 10.1007/s10236-016-0971-x (2016).

[8] Ewing, M. & Worzel, J. L. Long range sound transmission. *Geol. Soc. Am. Memo* **27** (1948).

[9] Urick, R. J. *Principles of Underwater Sound*. Peninsula Publishing, Page 151 (1983).

[10] Chu, P. C. & Fan, C. W. Exponential leap-forward gradient scheme for determining the isothermal layer depth from profile data. *Journal of Oceanography,* **73**, 503–526 (2017).

[11] Chu, P. C. & Fan, C. W. Global climatological data of ocean thermohaline parameters derived from WOA18. *Scientific Data*10.1038/s41597-023-02308-7 (2023). 37355660 10.1038/s41597-023-02308-7 PMC10290708

[12] Chu, P. C. & Fan, C. W. Global Ocean Climatological Dataset of 17 Thermohaline Parameters Derived from the World Ocean Atlas 2023. *NOAA National Centers for Environmental Information*10.25921/j3v2-jy50 (2024).

[13] Chu, P. C. & Fan, C. W. Global climatology of 13 underwater acoustic parameters derived from WOA-2023. *NOAA National Centers for Environmental Information*10.25921/fe7c-5f76 (2024).

[14] Chu, P. C. & Fan, C. W. A conserved minimal adjustment scheme for stabilization of hydrographic profiles. *Journal of Atmospheric and Oceanic Technology* **27**, 1072–1083 (2010).

[15] Chu, P. C. & Fan, C. W. Optimal linear fitting for objective determination of ocean mixed layer depth from glider profiles. *Journal of Atmospheric and Oceanic Technology* **27**, 1893–1898 (2010).

[16] Chu, P. C. & Fan, C. W. Maximum angle method for determining mixed layer depth from seaglider data. *Journal of Oceanography,* **67**, 219–230 (2011).

